# Sweet Basil Has Distinct Synthases for Eugenol Biosynthesis in Glandular Trichomes and Roots with Different Regulatory Mechanisms

**DOI:** 10.3390/ijms22020681

**Published:** 2021-01-12

**Authors:** Vaishnavi Amarr Reddy, Chunhong Li, Kumar Nadimuthu, Jessica Gambino Tjhang, In-Cheol Jang, Sarojam Rajani

**Affiliations:** 1 Temasek Life Sciences Laboratory, 1 Research Link, National University of Singapore, Singapore 117604, Singapore; vaishnavi@tll.org.sg (V.A.R.); chunhong@tll.org.sg (C.L.); kumarnadi@tll.org.sg (K.N.); jessicagambino@gmail.com (J.G.T.); jangi@tll.org.sg (I.-C.J.); 2 Department of Biological Sciences, National University of Singapore, Singapore 117543, Singapore

**Keywords:** eugenol synthase, phenylpropene, post-translational modifications, phosphorylation, secondary metabolism, sweet basil

## Abstract

Production of a volatile phenylpropene; eugenol in sweet basil is mostly associated with peltate glandular trichomes (PGTs) found aerially. Currently only one eugenol synthase (EGS), ObEGS1 which belongs to PIP family is identified from sweet basil PGTs. Reports of the presence of eugenol in roots led us to analyse other EGSs in roots. We screened for all the PIP family reductase transcripts from the RNA-Seq data. In vivo functional characterization of all the genes in *E. coli* showed their ability to produce eugenol and were termed as *ObEGS2-8*. Among all, *ObEGS1* displayed highest expression in PGTs and *ObEGS4* in roots. Further, eugenol was produced only in the roots of soil-grown plants, but not in roots of aseptically-grown plants. Interestingly, eugenol production could be induced in roots of aseptically-grown plants under elicitation suggesting that eugenol production might occur as a result of environmental cues in roots. The presence of *ObEGS4* transcript and protein in aseptically-grown plants indicated towards post-translational modifications (PTMs) of ObEGS4. Bioinformatics analysis showed possibility of phosphorylation in ObEGS4 which was further confirmed by in vitro experiment. Our study reveals the presence of multiple eugenol synthases in sweet basil and provides new insights into their diversity and tissue specific regulation.

## 1. Introduction

Volatile organic compounds (VOCs) produced by plants as part of their secondary metabolism are critical to various biological processes, which includes defence mechanism, protection from ultraviolet irradiation, chemical signalling, plant-plant interactions and plant-environment interactions [1,2]. VOC emissions from plants can be constitutive or induced as a response to abiotic or biotic stresses [3,4,5,6,7]. It has been reported that accumulation of VOCs and expression of pathway genes in plants is organ- or tissue- specific or developmental stage specific [8,9]. In recent times, a lot of progress with regards to elucidation of pathways leading to the formation of various plant VOCs has been made but information regarding the regulation of these specialized pathway especially under stress remains limited. Emerging research has shown that regulations can happen at a transcription level, translational level or post-translational level resulting in cell type specific stress response. Most of the studies on plant VOCs have focussed on emissions from aerial organs, but recent research shows that root produced VOCs play important and diverse roles in the rhizosphere. Root VOCs can affect microbial activity around it [10], alter behaviour of insects [11,12] and mediate belowground plant to plant communications [13].

**Figure 1:**
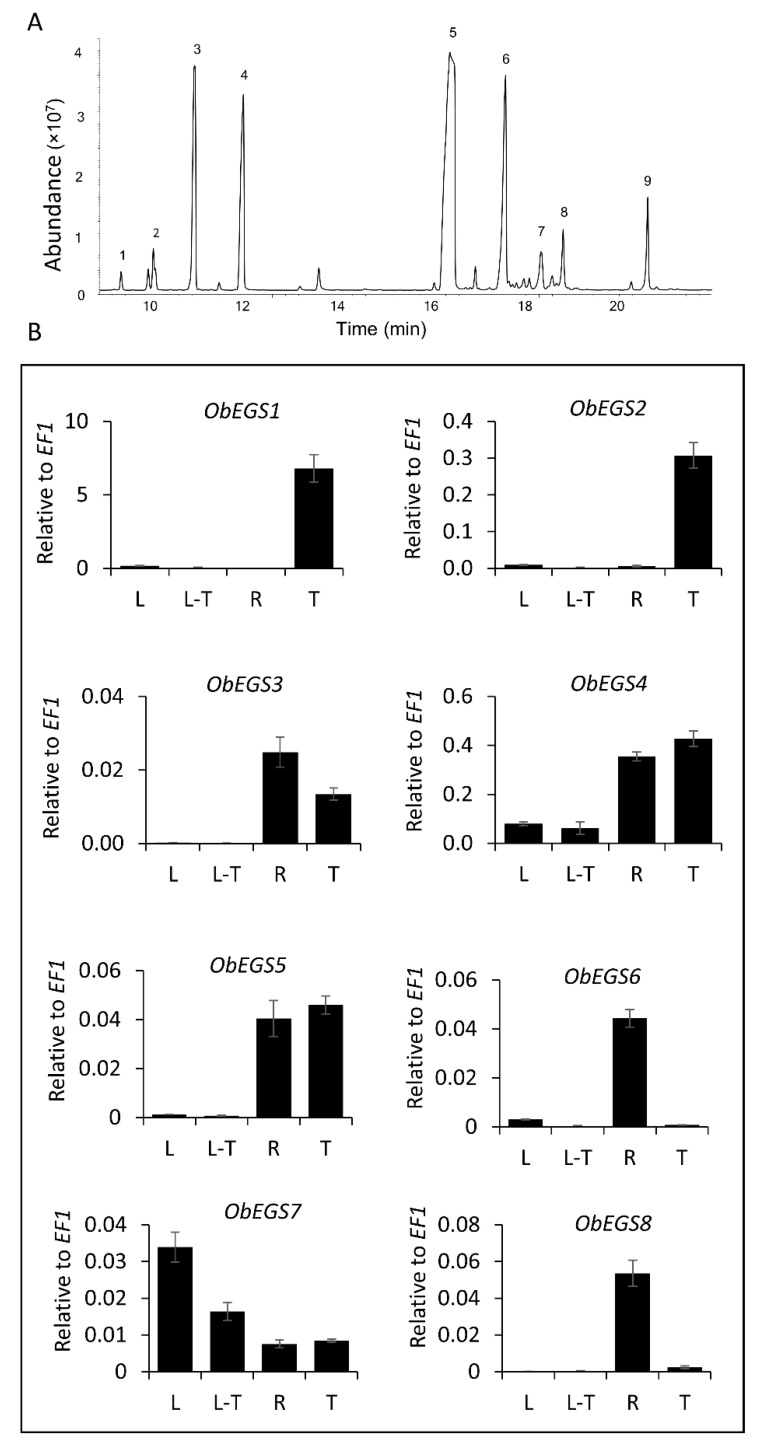
(**A**) GC-MS profile of sweet basil leaves. 1. alpha-pinene; 2. beta-pinene; 3. eucalyptol; 4. linalyl acetate; 5. eugenol; 6. alpha-bergamotene; 7. germacrene D; 8. gamma-muurolene; 9. β-copaene. (**B**) Expression levels of *ObEGSs* along various tissues. qRT PCR was done to analyse the expression of *ObEGSs* along the various tissues [leaf (L), leaf stripped of PGTs (L-T), root (R) and PGTs (T)]. *ObEGSs* showed varied expression along all four tissues. Error bars illustrate the SE of mean values.

*Ocimum* species produce and store a range of volatile phenylpropenes in specialized organs known as peltate glandular trichomes (PGTs), which are found on the aerial parts of the plant. Predominantly produced phenylpropenes in sweet basil varieties (*Ocimum basilicum*) are eugenol, chavicol and their methylated derivatives. In addition, they also make few terpenoids which includes eucalyptol, linalool and alpha-bergamotene [14,15] (Figure 1A). The first committed step of phenylpropene biosynthesis is catalysed by an acyltransferase, belonging to BAHD (Benzyl alcohol *O*-acetyltransferase, anthocyanin *O*-hydroxycinnamoyltransferase, *N*-hydroxycinnamoyl/benzoyltransferase, deacetylvindoline 4-*O*-acetyltransferase) family which acetylates monolignols, *p*-coumaryl and coniferyl alcohols to form *p*-coumaryl and coniferyl acetates respectively. These are acted on by phenylpropene synthases to produce different phenylpropenes (Supplementary Figure S1). These phenylpropene synthases can be allylphenol synthases (APS), which produce chavicol/eugenol or propenylphenol synthases (PPS) which produce *p*-anol/isoeugenol [16]. All phenylpropene synthases identified are NADPH-dependent aromatic alcohol reductases belonging to the PIP family, named after the first three identified members, pinoresinol-lariciresinol reductase (PLR) [17], isoflavone reductase (IFR) [18], and phenylcoumaran benzylic ether reductase (PCBER) [17].

In sweet basil PGTs, eugenol is produced from coniferyl acetate in a reaction catalysed by a phenylpropene synthase named eugenol synthase 1 (ObEGS1), which was previously identified from the EST collections constructed from basil glands and petunia flowers [14,19]. The substrate, coniferyl acetate, is formed from coniferyl alcohol by the action of BAHD family coniferyl alcohol acetyltransferase (CAAT). The first functionally characterized CAAT was PhCFAT from petunia [20]. Recently, two CAATs, ObCAAT1 and ObCAAT2 (PhCFAT homologue) were identified and characterized from sweet basil involved in eugenol synthesis [21]. Apart from ObEGS1, EGSs have been characterized from few other plants also such as Petunia (PhEGS1) [19], *Gymnadenia odoratissima* (GoEGS1 and GoEGS2) [22], *Clarkia breweri* (CbEGS1 and CbEGS2) [23], rose (RcEGS1) [24], strawberry (FaEGS1a and FaEGS1b) [25], and carrot (DcE(I)GS1) [26]. In the majority of these plants, EGS was shown to act only on coniferyl acetate to form eugenol, but in a few plants like in *Larrea tridentata* (LtCES1), EGS could act on both coniferyl acetate and *p*-coumaryl acetate as substrates to produce eugenol, and chavicol, respectively [27]. *EGSs* were also cloned from leaf tissues of four different *Ocimum* species using the previously identified *ObEGS1* sequence. These EGSs were shown to use coniferyl acetate as the preferred substrate for the biosynthesis of eugenol [28].

A recent study on eugenol biosynthesis in different tissues of sweet basil showed presence of eugenol in roots which lack PGTs [28]. Currently, all genetic studies involving eugenol production in sweet basil have been based on the aerial PGTs which constitutively produce eugenol. Although EGSs have been characterized from few plants, information on whether plants have distinct synthases for organ specific production of eugenol and how they get regulated by external factors is limited. To gain a comprehensive knowledge about eugenol production in sweet basil and eugenol synthases involved in its synthesis in PGTs and roots, a functional genomics approach was pursued. In our lab we have previously performed RNA-Seq of four tissues of sweet basil [leaf (L), leaf stripped of PGTs (L-T), roots (R) and PGTs (T)] [21]. The RNA seq data showed the absence of *ObEGS1* expression in roots. To identify possible eugenol synthases responsible for eugenol production in roots, we screened for all PIP family reductase transcripts and identified a total of seven PIP family reductase transcripts exhibiting expression in roots. We also checked for all PIP family reductase transcripts expressing in PGTs, and apart form ObEGS1, six PIP family reductase transcripts were also found expressed in the PGTs. In total 8 PIP family reductase transcripts including *ObEGS1* were identified from both PGTs and roots. Expression levels of the individual transcripts varied with majority showing expression in both PGTs and roots, which was further confirmed by quantitative PCR (q-PCR). *E. coli* in vivo functional characterization of all PIP family reductase transcripts including ObEGS1 demonstrated their ability to produce eugenol from coniferyl acetate and were termed as ObEGS2-8. This indicates that the roots of sweet basil which are devoid of PGTs harbour EGSs to produce eugenol. With respect to expression levels, *ObEGS1* had highest expression in PGTs and *ObEGS4* in roots. Interestingly, previous studies with respect to hairy root culture of sweet basil varieties have always been associated with rosmarinic acid (RA) production and not eugenol [29]. This hinted towards a different regulation of eugenol biosynthesis in roots of soil-grown and aseptically-grown roots. We propagated sweet basil plants in tissue culture medium and found that eugenol was not detected in the roots but was present in the leaves. Rosmarinic acid was found to be present in roots of tissue culture plants in much higher amounts than soil-grown plants. Quantitative RT PCR (qRT-PCR) showed the expression of all the *ObEGS*2-8 RNA in the sterile roots. In roots, *ObEGS4* had the highest expression, possibly it is the main contributor towards eugenol biosynthesis in roots, antibodies against ObEGS4 was raised to check for the presence of protein in the roots of aseptically-grown plants. Western blot showed the presence of ObEGS4 protein, alluding towards the possible role of post-translational modifications (PTMs) in the regulation of eugenol synthesis in roots. Elicitors are chemical compounds that can trigger stress responses in plants and plant cell cultures and, elicitor induced production of plant secondary metabolites is well known [30]. The application of elicitors could produce eugenol in the roots of aseptically-grown plants. PTM studies indicated that ObEGS4 is phosphorylated in aseptic conditions. The synthesis of specific secondary metabolites in plants helps them to adapt to various stress conditions in their growing environment. Growth in soil places the roots in a different ecological environment than tissue culture medium, where probably interaction with microbes or other factors requires the production of eugenol for successful establishment. This work has uncovered the gene family of EGSs in sweet basil, which are differentially expressed in PGTs and roots, expanding our knowledge about the diversity and evolution of enzymes involved in phenylpropene biosynthesis and provide new insights into the regulation of VOCs’ forming enzymes in plants.

## 2. Results

### 2.1. Identification of Eight Distinct Differentially Expressed PIP Family Reductases from Sweet Basil

**Figure 2:**
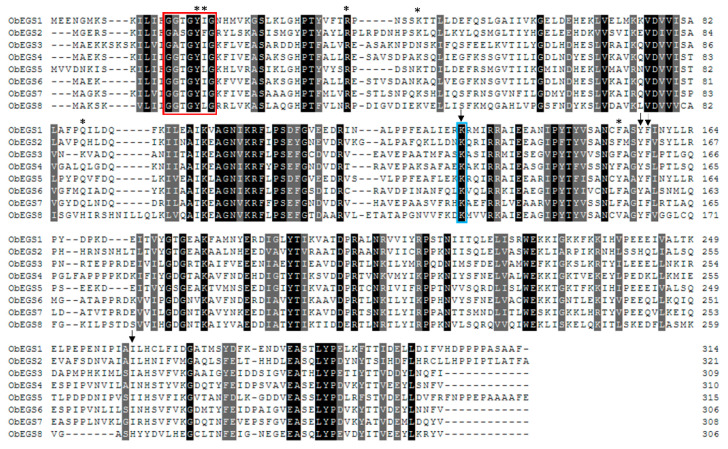
Clustal alignment of ObEGSs. All EGSs have a NAD(P) binding site G[GA]XGXXG which is highlighted with a red box. Active sites identified previously in ObEGS1 are indicated with * symbol. The arrows indicate key invariant residues in the previously published EGSs. Blue box shows the conserved lysine residue which has a catalytic role.

From the transcriptome data of sweet basil PGTs and roots, a total of eight PIP family reductase transcripts including ObEGS1 were identified and designated as *ObEGS1-8* (Figure 1B). Full-length cDNA of all eight transcripts were obtained. *ObEGS1* was previously reported [19], whereas *ObEGS2-8* which encoded 321, 309, 310, 315, 306, 308 and 306 amino acids respectively were newly identified in this study (Figure 2). The amino acid sequences of all eight EGSs were quite distinct from each other. On comparison with ObEGS1, ObEGS2-8 showed ~52%, 43%, 46%, 67%, 46%, 42% and 42% identity respectively (Supplementary Table S2). Characteristic domain of EGSs, NAD(P) binding site G[GA]XGXXG, was highly conserved in all ObEGSs. qRT-PCR was performed to validate the RNA-Seq data. In PGTs *ObEGS1* showed the highest expression whereas in roots *ObEGS4* showed highest expression (Figure 1B). *ObEGS2* had preferential expression in PGTs while *ObEGS6* and *ObEGS8* had preferential expression in roots. *ObEGS3* and *ObEGS5* had preferential expression in both, PGTs and roots while ObEGS7 had differential expression along all tissues tested.

**Figure 3:**
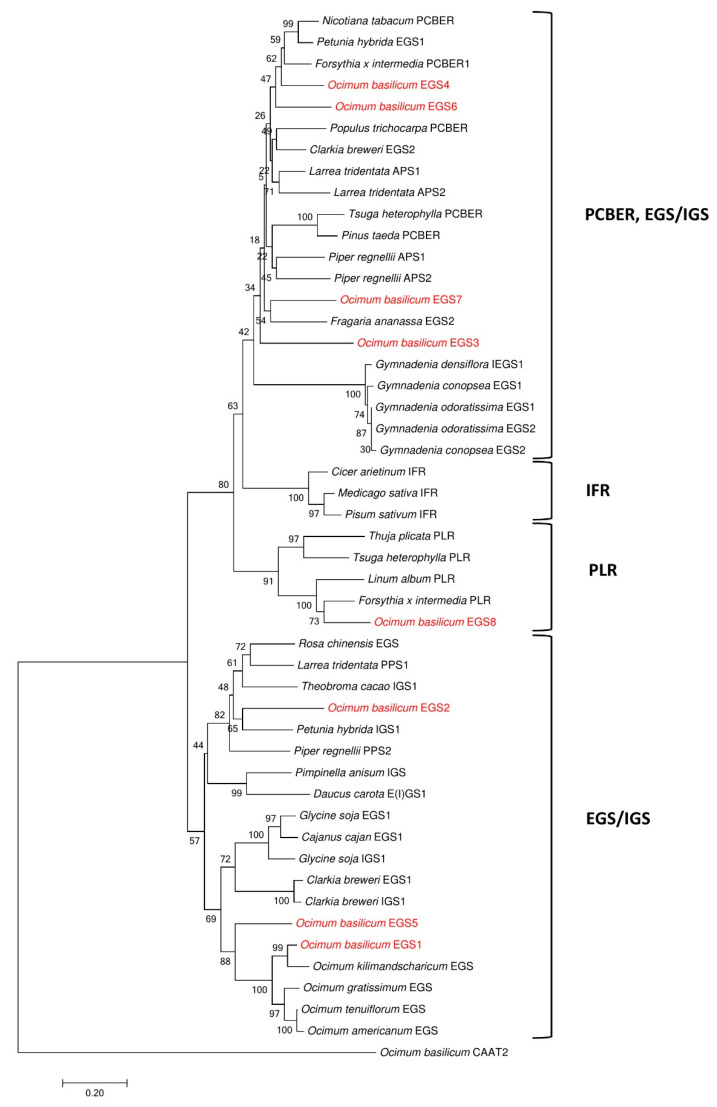
Phylogenetic tree showing the similarity of ObEGSs to known PIP family reductases. Tree was constructed using MEGA7 software by Neighbour-joining method with bootstrap values of 1000 replicates. The scale bar indicates the number of amino acid substitutions per site. ObCAAT2 which is unrelated to PIP family was used as an outgroup. EGS, eugenol synthase; IGS, isoeugenol synthase; PPS, propenyl-phenylpropene synthase; APS, allyl-phenylpropene synthase; PCBER, phenylcoumaran benzylic ether reductases; IFR, isoflavone reductases; PLR, pinoresinol-lariciresinol reductases.

A phylogenetic tree was constructed using the amino acid sequences of known PIP family of NADPH-dependent reductases from several other plants. ObCAAT2 which is unrelated to PIP family was used as an outgroup. The tree was divided into four groups, PCBER and EGS/IGS, IFR, PLR and EGS/IGS groups. ObEGS1, ObEGS2 and ObEGS5 fell under the EGS/IGS group while ObEGS3, ObEGS4, ObEGS6 and ObEGS7 fell under the PCBER and EGS/IGS group (Figure 3). ObEGS8 grouped with other PLR proteins suggesting it not be a true EGS. These results suggest that ObEGS1/2/5 and ObEGS3/4/6/7 may have distinct functions in different tissues.

**Figure 4:**
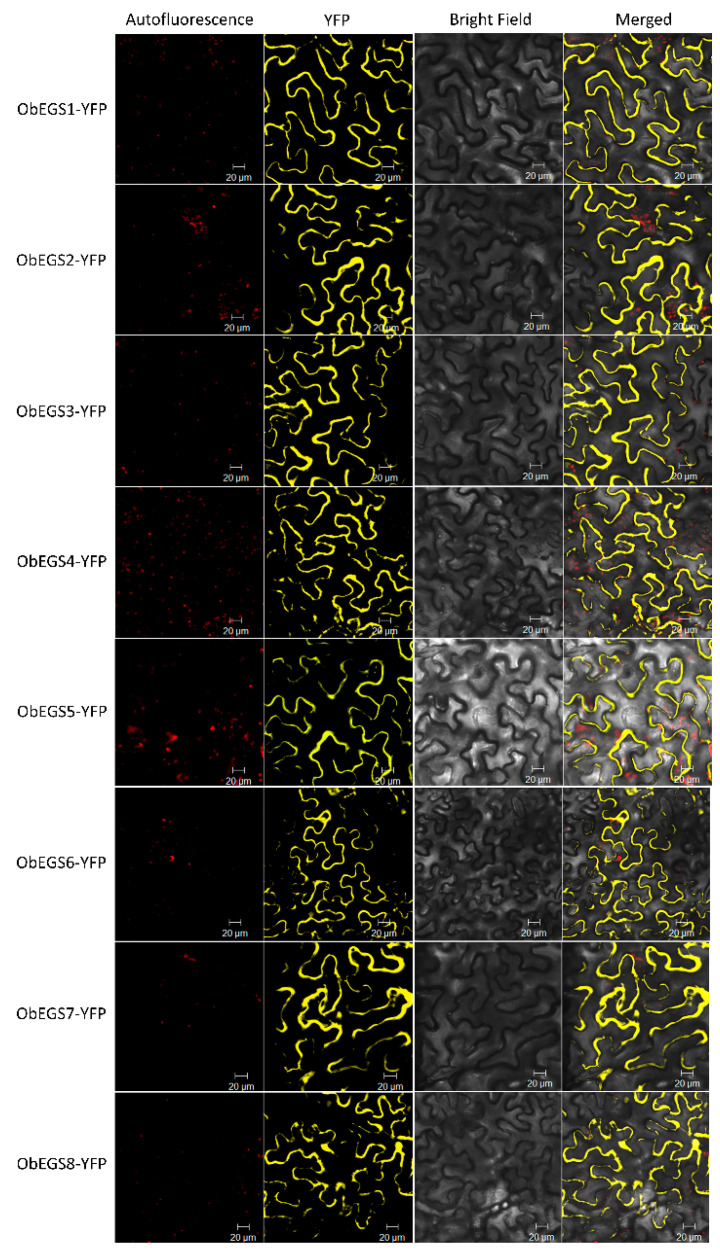
Subcellular localization of ObEGSs showing cytosolic localization in *N. benthamiana* leaf cells. *Agrobacterium*-mediated infiltration of YFP-tagged ObEGSs constructs were done in *N. benthamiana* leaf cells. YFP channel of a confocal microscope was used to visualize the cells 2 dpi. Autofluorescence: Chlorophyll autofluorescence channel image; YFP: YFP channel image; BRIGHT FIELD: light microscope image; MERGE: merged image between autofluorescence, YFP and light channel.

To examine subcellular localization of EGSs, the open reading frames of EGSs were fused with 5′-terminus of YFP and expressed under the control of CaMV 35S promoter. The recombinant constructs were then introduced into *N. benthamiana* leaves by agroinfiltration. All eight ObEGSs were localized in the cytosol (Figure 4). Sweet basil PGTs where eugenol is mainly produced are highly cytoplasmic [14] and monolignols, which are shared substrates for phenylpropene and lignin production [31] are known to be synthesized in cytosol [32].

### 2.2. Functional Characterization of EGSs

**Figure 5:**
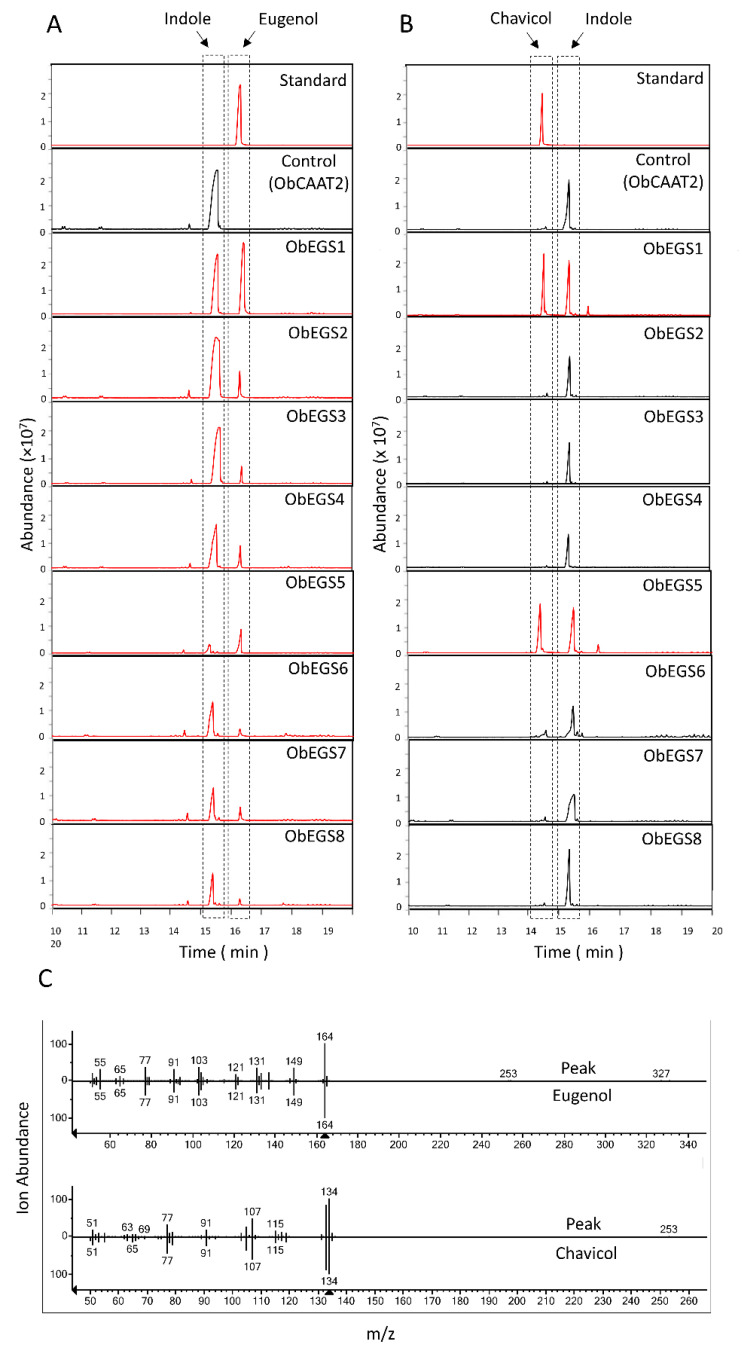
GC-MS analysis of products formed in *E. coli* spent medium by ObEGSs. (**A**) Eugenol was produced by all eight EGSs using coniferyl alcohol as the substrate, while (**B**) chavicol was produced only by ObEGS1 and ObEGS5 from *p*-coumaryl alcohol. (**C**) Mass spectra of the obtained eugenol and chavicol peak compared to the matched eugenol and chavicol peak from the NIST library are shown at the bottom of chromatograms. ObCAAT2 served as negative control. Indole peak was seen in all bacterial samples. Red chromatograms indicate presence of eugenol or chavicol peak.

To characterize the enzyme activity of EGSs, we used *E. coli* cultures harbouring ObEGSs using *p*-coumaryl alcohol and coniferyl alcohol as substrates. Coniferyl alcohol can be acylated to coniferyl acetate by endogenous acyltransferases in *E. coli* [19], suggesting that *p*-coumaryl alcohol could also be converted into *p*-coumaryl acetate in *E. coli*. Figure 5A,B shows that all eight EGSs with coniferyl alcohol as substrate produced eugenol whereas only ObEGS1 and ObEGS5 with *p*-coumaryl alcohol as substrate showed chavicol production. This was in accordance to previous study showing that ObaI EGS which is identical to ObEGS1 has the dual function to produce eugenol and chavicol from coniferyl alcohol, and *p*-coumaryl alcohol, respectively [28]. However, ObEGS2/3/4/6/7/8 were able to produce only eugenol and not chavicol. *E. coli* cultures harbouring ObCAAT2 served as a negative control which produced only indole, which is a volatile naturally produced by *E. coli* cells. Mass spectra of resulting peaks were confirmed with those of NIST library as well as authentic standards (Figure 5C).

### 2.3. Transient Expression of EGSs in Tobacco Plants

**Figure 6:**
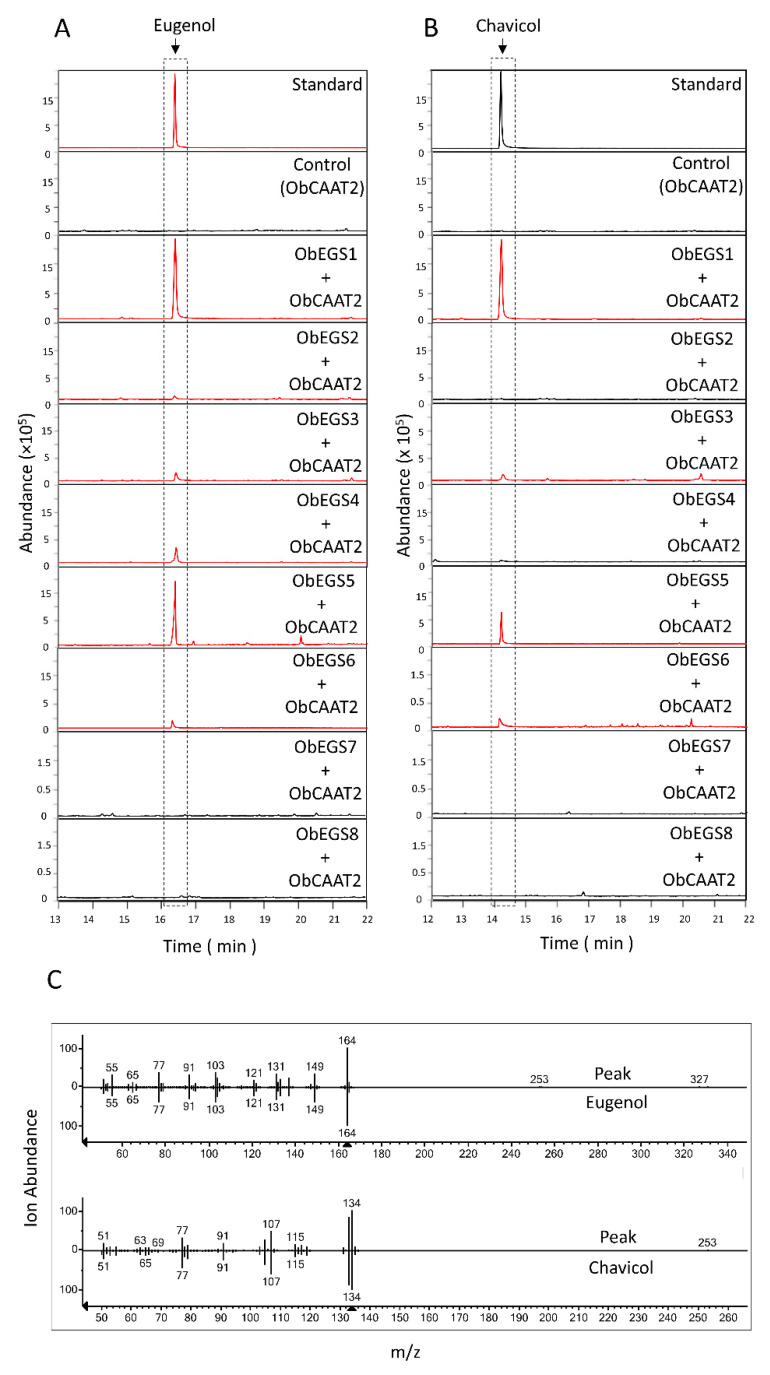
GC-MS analysis of products formed *in planta* by ObEGSs. (**A**) Only ObEGS1-6 produced eugenol from coniferyl alcohol however ObEGS1 produced more compared to other EGSs. (**B**) Only ObEGS1, ObEGS3, ObEGS5 and ObEGS6 produced chavicol from *p*-coumaryl alcohol. (**C**) Mass spectra of the obtained eugenol and chavicol peak compared to the matched eugenol and chavicol peak from the NIST library are shown at the bottom of chromatograms. ObCAAT2 served as negative control. Red chromatograms indicate presence of eugenol or chavicol peak.

To characterize the ObEGSs in planta, *Agrobacterium* cultures containing the plasmids expressing each *ObEGS* under CaMV35S promoter were used to infiltrate the leaves of *N. benthamiana* together with or without *35S_pro_:ObCAAT2. N. benthamiana* plants were used because they can produce high yields of protein in a relatively short period of time (2–3 days). After 3 d (days), the volatiles emitted out of the plant were trapped using a headspace and analysed using GC-MS. However, no peaks of eugenol or chavicol was observed. Later, the infiltrated plants were again re-infiltrated with the substrates, *p*-coumaryl alcohol or coniferyl alcohol, and analysed using GC-MS. As shown in Figure 6A,B, ObEGS1-6 produced eugenol with coniferyl alcohol as substrate while ObEGS1, ObEGS3, ObEGS5 and ObEGS6 were able to produce chavicol, as well with *p*-coumaryl alcohol as substrate. Under in planta conditions, ObEGS7 and ObEGS8 were not able to produce significant peaks of eugenol or chavicol with coniferyl alcohol or *p*-coumaryl alcohol respectively but under *E. coli* in vivo conditions minute amounts of eugenol production was observed suggesting that the two different assay conditions has an effect on their enzymatic activity. Mass spectra of resulting peaks were confirmed with those of NIST library as well as authentic standards (Figure 6C). In planta assays with ObCAAT2 was used as a negative control.

### 2.4. Analysis of ObEGS1 and ObEGS4 Promoter Shows the Presence of Different Cis Regulatory Elements

**Figure 7:**
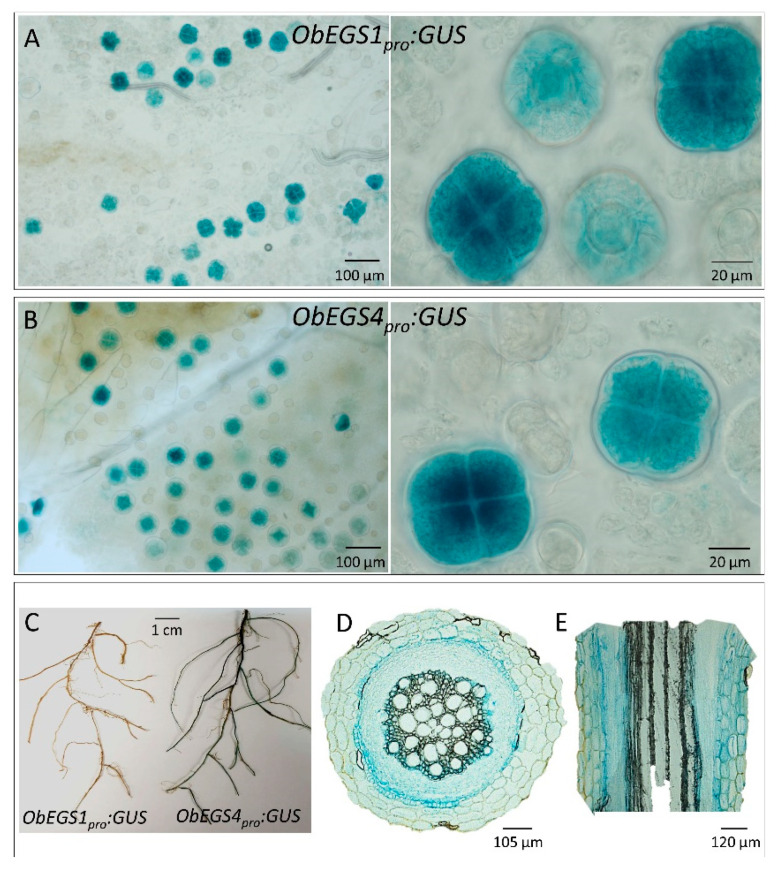
Expression pattern of *ObEGS1* and *ObEGS4* promoter in sweet basil. (**A**) PGTs-specific expression of *ObEGS1_pro_:GUS* on sweet basil leaf surface. (**B**) PGTs-specific expression of *ObEGS4_pro_:GUS* on sweet basil leaf surface. (**C**) Roots of *ObEGS4_pro_:GUS* transformed sweet basil roots showing GUS expression. (**D**) Transverse and (**E**) longitudinal sections of GUS stained roots showing GUS expression in non-vasculature tissues.

*ObEGS1* and *ObEGS4*, which are highly expressed in PGTs, and roots, respectively were selected for promoter analysis to gain insights into gene regulation. The 2027-bp *ObEGS1* and 2149-bp *ObEGS4* genomic DNA fragments upstream of the translation start site was cloned by genome walking. Apart from the common CAAT and TATA box, several other *cis*-acting regulatory elements were identified by bioinformatics analysis using PlantCARE tool (http://bioinformatics.psb.ugent.be/webtools/plantcare/html/). These elements are shown in Supplementary Tables S4 and S5. Interestingly an AC-II element was found in *ObEGS1* promoter and a MYB biding site (MBS) was found in *ObEGS4*. In general, AC-rich regions and MBS are known to be bound by R2R3-MYBs [33,34]. This suggests that *ObEGS1* and *ObEGS4* might be regulated by R2R3-MYBs. Previous studies have reported involvement of an R2R3-MYB in regulation of eugenol production in ripe strawberry fruit receptacles [35]. Also, the presence of several elements for hormones and plant defence in *ObEGS4* promoter suggest differential regulation of *ObEGS4* under the influence of hormones or for plant defence when compared to *ObEGS1*. To check for the expression pattern, the promoter fragments were cloned to drive β-glucuronidase (GUS) reporter gene and transformed into sweet basil plants. Leaves of transgenic plants of *ObEGS1*_pro_:GUS and *ObEGS4*_pro_*:GUS* plants showed PGT-specific staining (Figure 7A,B). In addition, *ObEGS4*_pro_*:GUS* plants showed GUS staining in roots mainly in non-vascular cells. (Figure 7C–E).

### 2.5. Eugenol Was Not Observed in Roots of Aseptically-Grown Plants but Could Be Induced by Elicitors

**Figure 8:**
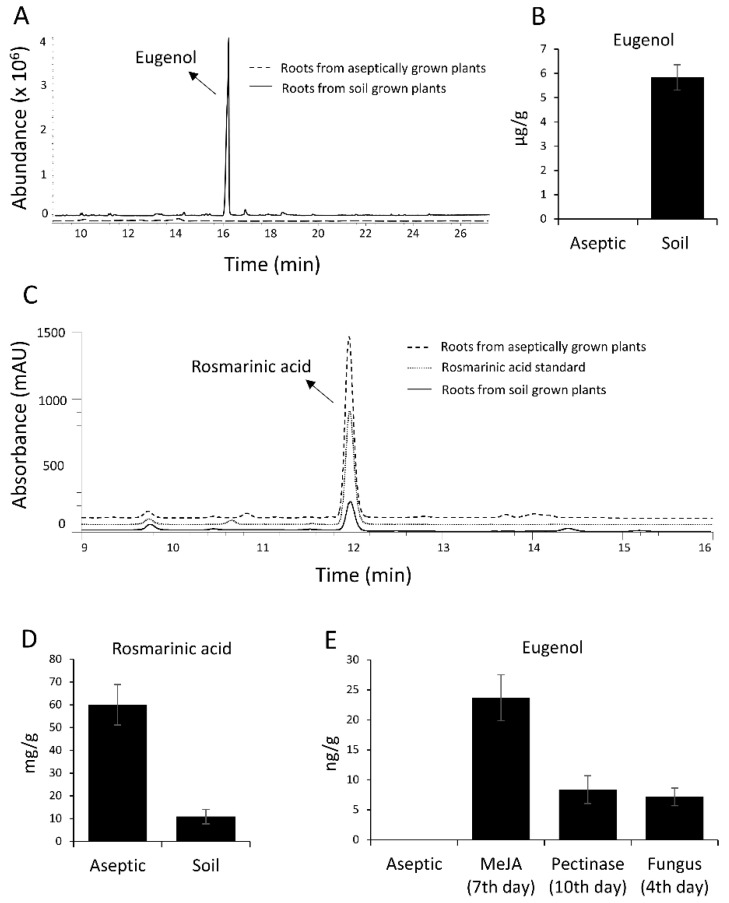
GC-MS and HPLC profiles of eugenol and rosmarinic acid in roots of soil-grown plants and aseptically-grown plants. (**A**) GC-MS profile of roots of soil-grown plants showing a peak of eugenol which is absent in aseptically-grown plants. (**B**) Eugenol was undetectable in roots of aseptically-grown plants whereas ~ 6 µg/g of eugenol was found in roots of soil-grown plants. (**C**) HPLC profile of of roots of soil-grown plants and aseptically-grown plants. (**D**) Amount of rosmarinic acid was more in roots from aseptically-grown plants when compared to roots from soil-grown plants. (**E**) Eugenol was undetectable in roots of aseptically-grown plants however under stress conditions ~10–22 ng/g of eugenol was detected.

**Figure 9:**
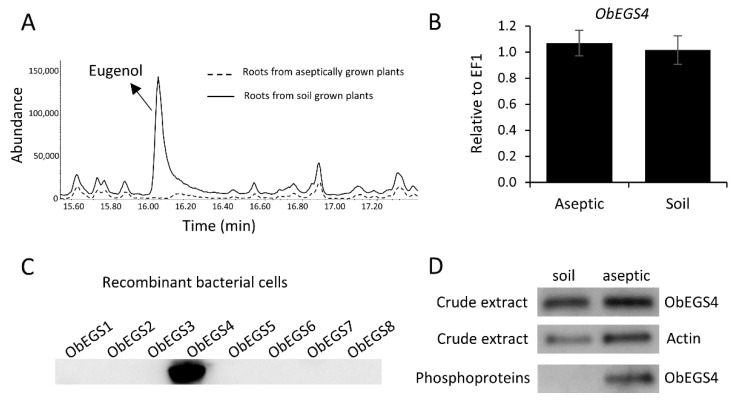
Expression analysis of ObEGS4 in soil-grown roots and aseptically-grown roots. (**A**) GC-MS profile of processed roots for detecting glycosylated eugenol. Soil-grown plants show a peak of eugenol which is absent in aseptically-grown plants. (**B**) mRNA expression of ObEGS4 transcript was detected in both kinds of roots. (**C**) Custom made ObEGS4 antibody specifically binds only to ObEGS4 protein. (**D**) ObEGS4 protein is present only in the pool of phosphoproteins from aseptically-grown roots.

The presence of eugenol was examined in soil-grown and aseptically-grown sweet basil plants by GC-MS analysis. Eugenol was found in the leaves of both soil-grown and aseptically-grown plants but only roots of soil-grown plants showed the presence of eugenol, which was not detected in the roots of plants grown in aseptic conditions (Figure 8A,B). Often in plants, volatiles, such as eugenol can be glycosylated and stored in vacuoles to increase stability and reduce toxicity [36,37]. To test whether eugenol is being stored in a glycosylated form in aseptically-grown conditions, the root samples were processed to convert any possible glycosylated eugenol present into their aglycones, which then can be identified using GC-MS. However, eugenol was still not detected negating the possibility of presence of eugenol in glycosylated form in aseptically-grown roots (Figure 9A).

In order to test whether eugenol production in roots can be triggered by biotic or abiotic stresses, 45 d old aseptically-grown sweet basil plants were treated with five different elicitors, gibberellic acid (GA), salicylic acid (SA), methyl jasmonate (MeJA), ethylene and pectinase as well as bacteria and fungus. As shown in Figure 8E, eugenol was detected in roots of aseptically-grown plants grown in plates containing MeJA and pectinase when subjected to GC-MS on 7th day and 10th day respectively. GA, SA and ethylene did not show any effect on the production of eugenol. Bacterial infection did not show any induction however fungal infection induced eugenol production which was detected on fourth day post-infection. However, the amount of eugenol produced in roots of aseptically-grown plants under elicitation was much lower than the amount produced in roots of soil-grown plants. This might be because of the different external factors and multiple stress conditions experienced in soil as compared to aseptic conditions. Previous studies have shown production of rosmarinic acid in sweet basil hairy roots [29], and thus, we analysed rosmarinic acid production in our plant roots using HPLC (Figure 8C) and estimated the amount produced in roots of aseptically-grown plants and soil-grown plants. Amount of RA was more in aseptically-grown roots as compared to soil-grown roots (Figure 8D).

### 2.6. PTMs Can Regulate Eugenol Synthase in the Roots of Sweet Basil

To understand the environmental regulation of eugenol synthase in roots, the expression levels of all *ObEGSs* were analysed in roots of aseptically-grown plants and soil-grown plants. All the *ObEGSs* were found expressed in both kinds of roots, suggesting that *ObEGSs* might not be regulated at transcriptional level in aseptically-grown plants (Figure 9B) (Supplementary Figure S3). To analyse whether ObEGSs are regulated translationally in roots, we studied ObEGS4 as it has the highest expression in roots when compared to others. To test the expression of ObEGS4 protein, a custom-made antibody was raised against a 14 amino acid sequence of ObEGS4 (PAKSAFAEKAKIRR). ObEGS4 antibody was then tested to confirm its specificity to ObEGS4 (Figure 9C). Western blot analysis detected the presence of ObEGS4 protein in both, soil-grown and aseptically-grown roots indicating that the ObEGS4 is not regulated at translational level (Figure 9D). To investigate the possibility of any possible PTMs of ObEGS4, several bioinformatic analysis were pursued. Using NetPhos 3.1 [38] several potential sites for phosphorylation were identified (Supplementary Figure S2). A high threshold (≥0.90) was set to increase the accuracy of prediction. To validate the bioinformatic prediction, phosphoproteins were enriched from both kinds of roots and tested against the ObEGS4 antibody. Interestingly, we found that the ObEGS4 was found phosphorylated in roots grown aseptically, but not in roots grown under soil conditions. However, phosphorylation might be one of several PTMs which might be involved in this PTM based regulation of ObEGS4. We also looked for potential sites for ubiquitination using the UbSite tool [39], but no potential sites were observed. To validate this, ubiquitinated proteins were enriched and tested against ObEGS4 antibody. The absence of ObEGS4 bands indicated that ObEGS4 does not undergo ubiquitination. In addition, the expression of ObCAAT2 was also analysed in aseptically grown roots which is involved in the production of substrate for EGSs and was found to be expressed (Supplementary Figure S3).

## 3. Discussion

Plant secondary metabolites are important for plants’ fitness and adaption to the ever-changing biotic and abiotic environment. Many of these metabolites are biochemically expensive to produce, hence their production is tightly regulated at multiple levels to ensure synthesis is in a tissue specific manner or in response to specific ecological conditions. Great advances have been made in elucidating the pathway genes involved in the formation of many of these secondary metabolites but full understanding of the complex regulatory mechanism behind these pathways remains limited. The role of transcription factors, miRNA, feedback mechanisms, post-transcriptional and post-translational regulation of pathway enzymes are all known to be involved in the regulation and optimization of the metabolic flux. Sweet basil produces a volatile phenylpropene, eugenol predominantly in PGTs found on the aerial parts of the plant. Eugenol produced by plants act as floral attractant of pollinators and as a defence compound [19]. It has also shown to possess several biological activities like antifungal, anti-inflammatory, antiviral, anticarcinogenic, antioxidant and antibacterial [40]. The final step in synthesis of eugenol is catalysed by eugenol synthase.

To date, only one EGS, ObEGS1, has been isolated and characterized from *O. basilicum* PGTs. A comprehensive analysis of RNA-Seq data from PGTs and roots of sweet basil identified seven new EGSs apart from ObEGS1. qRT-PCR analysis revealed varied expressions of *ObEGSs* in PGTs and roots. Cytosolic localization of all EGSs indicates that they are suitably localised to participate in the final steps of phenylpropanoid pathway. Although, all the EGSs were able to catalyse the same biochemical reaction in *E. coli*, their sequences are quite divergent. This suggests that each member of this family probably has its own range of substrates. Phylogenetic analyses showed that ObEGS1/2/5 group together in a clade while ObEGS3/4/6/7 group together in a different clade hinting towards different evolutionary pathways.

Studies in *E. coli* showed that only ObEGS1 and ObEGS5 could produce both eugenol and chavicol from coniferyl alcohol and *p*-coumaryl alcohol respectively. Whereas, ObEGS2, ObEGS3, ObEGS4, ObEGS6, ObEGS7 and ObEGS8 could produce eugenol from coniferyl alcohol but no production of chavicol was observed from *p*-coumaryl alcohol. In planta studies showed that ObEGS2 and ObEGS4 could only produce eugenol whereas ObEGS1, ObEGS3, ObEGS5 and ObEGS6 could produce both eugenol and chavicol from coniferyl alcohol, and *p*-coumaryl alcohol, respectively. However, compared to ObEGS1 and ObEGS5, the amount of chavicol produced by ObEGS3, and ObEGS6 was very low. The discrepancy can be due to the lack of additional cofactors required by ObEGS3 and ObEGS6 in *E. coli*, which are required for catalysing the chavicol reaction. In *E. coli* ObEGS7 and ObEGS8 could produce eugenol from coniferyl alcohol however in in planta no significant peaks of eugenol was observed. In *E. coli*, highly expressed enzymes with large amounts of substrate are used, which usually does not mimic the true complex plant cell background, in terms of limiting substrate, cellular activators and inhibitors which affects enzymatic activity. Phylogenetic analysis also revealed that ObEGS8 fell into the PLR clade indicating that it might not have an EGS activity in planta. Based on expression patterns and functional characterization, ObEGS1 is likely to contribute most towards eugenol production in PGTs and ObEGS4 in roots. Previous studies have demonstrated a strong correlation between EGSs expression pattern and amount of eugenol synthesized [25].

Plant roots are known to produce and release specialized metabolites, including volatile organic compounds into the rhizosphere which mediates an array of below ground communications. Some metabolites are constitutively released while others are induced by environmental cues. Emission of root volatiles as a stress response has been reported [41,42]. In sweet basil roots, eugenol production is observed only in plants grown in soil and not in aseptically-grown plants. Roots growing in soil are more prone to pathogens, temperature changes and other stresses that might require eugenol production for better fitness. In our study, MeJA could induce eugenol production in aseptically-grown roots along with pectinase and fungus. This infers that the substrates and enzymes for eugenol production are present in aseptically-grown roots but they need an activating signal to catalyse. MeJA is known to be an important signal in the regulation of plant responses to pathogens, wounding, temperature and salinity stress [43]. Eugenol production under fungal infection and pectinase treatment which mimics wounding might be a part of the biotic stress response of the plant. Eugenol is known to possess antimicrobial activity. Hence eugenol production in roots can be a defence mechanism against pathogenic microbes in soil. Apart from biotic stress, other abiotic stresses can also together contribute to production of eugenol in roots of soil-grown sweet basil plants. The precise biological benefit imparted by eugenol production in sweet basil roots remains to be elucidated. Additionally, RA was also detected in the sweet basil roots, but the amount was less in soil-grown roots when compared to aseptic conditions. This illustrates the fact that the quality and quantity of secondary metabolites profiles varies under different environmental conditions for providing better adaptability [44,45].

Many secondary metabolites including volatile organic compounds are known to undergo post-production glycosylation, which reduces their toxicity and enhances water solubility to enable storage in subcellular compartments. Such glycosylated compounds act as stored precursors for the production of aglycone under proper developmental or environmental cues [37]. However, absence of glycosylated eugenol in aseptically-grown plants suggests a different regulation of eugenol biosynthesis under aseptic conditions when compared with soil conditions. The expression of *ObEGS4* transcript and the presence of ObEGS4 protein negates the possibility of transcriptional, post-transcriptional and translational regulation of ObEGS4 in roots of aseptically-grown plants. Post-translational modifications are known to be involved in the regulation of numerous plant metabolic pathways. PTM allows for rapid changes in protein function in response to changes in environment. Phosphorylation and ubiquitination are among the several PTMs that play an important role in plant response to stress conditions [46]. The presence of phosphorylated ObEGS4 in aseptically-grown roots and not in soil-grown roots shows that ObEGS4 is post-translationally regulated, which might affect its activity. Previously it has been shown that enzymes in phenylpropanoid pathway of sweet basil can be post-translationally modified, which leads to lower levels of metabolite in spite of high levels of mRNA and protein [47]. In poplar, it was shown that phosphorylation of 5-hydroxyconiferaldehyde *O*-methyltransferase alters its activity negatively [48]. However, PTMs are not limited by phosphorylation and ubiquitination and there can be additional PTMs of ObEGS4 that needs to be deciphered. Therefore, the post-translational regulation of ObEGS4 might be a result of coordinated effort of multiple PTMs.

Apart from regulation of ObEGSs in roots, absence of eugenol in aseptically-grown roots can also be due to inactivity of upstream enzymes of eugenol pathway. The first committed step towards eugenol formation is catalysed by BAHD family CAAT, which converts coniferyl alcohol to coniferyl acetate. Coniferyl alcohol is also a substrate for lignin biosynthesis. Lignin is a key structural component of plant cell wall and vasculature, hence pathway leading up to coniferyl alcohol formation should be presumably active in aseptically-grown roots. Apart from *ObCAAT2*, RNA-seq data of root revealed several other BAHD family AAT transcripts like *ObCAAT2*. BAHD enzymes are known to display substrate versatility by accepting other alcohol substrates and thereby functioning in multiple pathways [21,49]. In aseptically grown roots expression of *ObCAAT2* is observed. Whether ObCAAT2 is the only enzyme responsible for the formation of coniferyl acetate in the roots and becomes phosphorylated in sterile conditions remains to be deciphered.

In conclusion, our study provides new insights on the regulation of eugenol production in roots under environmental cues. It also contributes towards understanding eugenol production in different tissues of the sweet basil by multiple ObEGSs. This will help to create new strategies to study plant defence mechanisms and to investigate how the biosynthetic enzymes involved in the secondary metabolism are regulated by environmental factors.

## 4. Materials and Methods

### 4.1. Plant Material and RNA Isolation

Commercial variety of sweet basil (*Ocimum basilicum*) plants were propagated from seeds and grown in greenhouse under Singapore’s natural conditions. PGTs were isolated from 3–4 cm leaves as described previously [50]. Total RNA was extracted from PGTs using the Spectrum Plant total RNA kit from Sigma (Singapore) according to manufacturer’s protocol.

### 4.2. Gene Amplification and Plasmid Construction

Full-length ORFs were obtained by performing 3′ and 5′ rapid amplification of cDNA ends (RACE) using the SMARTer TM RACE cDNA amplification kit from Clontech (Mountain View, CA, USA). ORFs were then inserted into pDEST vector and transformed into BL21 cells for *E. coli* assays. They were also inserted into pBADC vector and transformed into Agrobacterium EHA105 for in planta and localization studies.

### 4.3. Quantitative Real Time PCR (qRT-PCR)

Expression levels of *ObEGS1-8* along various tissues (leaf, leaf stripped of PGTs, root and PGTs) were analysed using qRT-PCR. Approximately 500 ng of RNA was reverse transcribed to cDNA using iScript^TM^ cDNA Synthesis kit from Bio-Rad (Singapore). The qRT-PCR reactions were performed in 384-well PCR plate using ABI PRISM 900HT real-time PCR system and KAPA SYBR fast master mix (KAPA Biosystems, Sigma, Singapore). A sum of 0.3 µL of cDNA was used for a total PCR reaction of 5 µL and cycling profile was 50 °C for 2 min, 95 °C for 10 min, 40 cycles of 95 °C for 15 s and 60 °C for 60 s. After thermal cycles, the dissociation analysis (melting curve) was carried out to confirm specific amplification of PCR reaction by adding a profile of 95 °C for 15 s, 60 °C for 15 s and 95 °C for 15 s. In current study, sweet basil elongation factor 1 (*ObEF1*) was used as an internal control, due to its stable expression in plant [51] and also it showed similar expression levels in all the tissues in the transcriptome data of sweet basil. Comparative delta C_T_ values of target genes to *ObEF1* were taken as relative expression among different tissues. The amount of target gene, normalized to *ObEF1* gene, was calculated by 2^−(C^_T_^target gene−C^_T_*^ef1^*^)^ [52]. Error bars represent mean ± SE which were calculate from three biological replicates each analysed in triplicates, including non-template control.

### 4.4. Subcellular Localization of ObEGSs

The full-length cDNAs of *ObEGS1-8* without the stop codon were cloned into the gateway vector pENTR/D-TOPO (Invitrogen, Darmstadt, Germany), and then subsequently transferred into the destination vector pBA-DC-YFP [53], which contains the cauliflower mosaic virus (CaMV) 35S promoter and yellow fluorescent protein (YFP) in frame at the C-terminal, to generate ObEGS1-YFP, ObEGS2-YFP, ObEGS3-YFP, ObEGS4-YFP, ObEGS5-YFP, ObEGS6-YFP, ObEGS7-YFP and ObEGS8-YFP respectively. The constructs were then introduced into *Agrobacterium tumefaciens* strain EHA105 by a heat shock method. Overnight cultures of *Agrobacterium* grown at 28 °C were harvested and resuspended to a final concentration of absorbance of 1.0 at 600 nm in a solution containing 10 mM MgCl_2_, 10 mM MES pH 5.6 and 100 µM acetosyringone. After 3 h incubation at room temperature, the *Agrobacterium* mixture was injected into *Nicotiana benthamiana* leaves using a needleless syringe. Infiltrated tobacco plants were placed in the growth chamber at 24 °C for 2 d. After 2 d, the fluorescence signals were detected by a confocal scanning laser microscopy (Carl Zeiss LSM 5 Exciter) with a standard filter set. All primers used in this study are listed in Supplementary Table S1.

### 4.5. Promoter Analysis

Genomic DNA was isolated from leaves of sweet basil plants using cetyl trimethylammonium bromide method. The flanking sequences of genes were amplified using a GenomeWalker™ Universal kit (Clontech, Mountain View, CA, USA) and later ligated to pGEM^®^-T vector. The resulting product was transformed into *E. coli* XL1-Blue cells and sequenced. The promoter was amplified with Phusion^®^ High-Fidelity DNA Polymerase (New England Biolabs, Beverly, MA, USA) and subcloned into a gateway donor vector pENTR™/D-TOPO^®^ (Invitrogen, Darmstadt, Germany). Further, the recombinant plasmid was introduced into destination vector pBGWFS7 by LR recombination. The destination plasmid was further transformed into *Agrobacterium* EHA105 by heat shock. The recombinant *Agrobacterium* EHA105 strain was used to generate transgenic sweet basil lines. Transformed plants were subjected to β-Glucuronidase (GUS) staining by dipping the tissues in GUS staining solution and incubating at 37 °C for overnight in the dark. On the next day, the tissues were cleared by soaking in ethanol to remove chlorophyll and the GUS-stained tissues were photographed using a Zeiss Whitefield microscope.

### 4.6. Histology

GUS-stained roots were fixed in 4% formaldehyde for 16 h followed by a series of dehydration with 20%, 40%, 60%, 80% and 100% ethanol for 1 h each. The roots were then left at 100% ethanol for overnight at 4 °C. Next day, the dehydrated roots were treated with 2:1 ratio of ethanol:infiltration medium (Leica, Chemoscience, Singapore) for 2 h, followed by 1:2 ratio of ethanol:infiltration medium for another 2 h. The roots were then left in 100% infiltration medium for overnight. Next day, the roots were embedded in the embedding medium (Leica, Chemoscience, Singapore) and left inside fume hood for 2 d. Finally, the embedded roots were mounted on to the holders which once dried were used for sectioning of the roots using microtome. The sections were then photographed using a Zeiss Whitefield microscope.

### 4.7. Sweet Basil Transformation

*Agrobacterium*-mediated transformation of sweet basil was done by the following procedure. *Agrobacterium* EHA105 cells transformed with desired construct were cultured in 15 mL LB liquid medium, containing antibiotics at 28 °C for 2 d. This culture was then used to inoculate 150 mL of LB medium with selected antibiotics and incubated at 28 °C, until OD_600_ reached 0.9. The cells were then pelleted and resuspended in 80 mL of LB medium containing acetosyringone (100 µM/L). This culture was used for transformation of sweet basil. 40% Clorox was used for sterilizing sweet basil seeds by washing for 3 min. Later the seeds were rinsed several times with sterile water. The sterile seeds were then imbibed at 4 °C overnight. The following day, to harvest the mature embryos, the seeds were dissected under a dissection microscope. The dissected embryos were precultured in dark for one day in Murashige and Skoog (MS) media plates. The precultured embryos were then immersed in *Agrobacterium* culture and sonicated for 15 s, four times. After sonication, the embryos were immersed in fresh *Agrobacterium* solution and vacuum infiltrated for 3 min. After infection, the embryos were placed in co-cultivation (CC) media plates [MS salts + 6-Benzylaminopurine (BA) (0.4 mg/L) + myo-inositol (100 mg/L) + cefotaxime (150 mg/L) + indole-3-butyric acid (0.4 mg/L) + sucrose (30 g/L)] for 3 d. Later, sterile distilled water containing cefotaxime (150 mg/L) was used to wash the embryos multiple times. The washed embryos were kept in CC media plates for 3-4 weeks in dark for shoot induction. The red fluorescent protein (RFP) was used as a visual selection marker. After 3–4 weeks RFP positive shoots were selected and transferred to light. The well grown shoots were transferred to elongation media plates [MS salts + cefotaxime (150 mg/L) + sucrose (30 g/L) + indole acetic acid (0.5 mg/L) + BA (3 mg/L)] and kept for 2–3 weeks. The shoots were hardened on basal media plates and allowed for root formation. Plantlets with well-developed roots were transferred to soil and grown under greenhouse conditions before further analysis. Plant culture room temperature was maintained at 24 °C and light conditions were 16 h light and 8 h dark.

### 4.8. In Vivo Assays in E. coli and Tobacco Leaves

For the *E. coli* in vivo feeding assay, 50 mL liquid cultures of *E. coli* harbouring ObEGSs expression constructs were induced with 0.5 mM isopropyl β-d-1-thiogalactopyranoside (IPTG), substrates at a final concentration of 100 µg/mL were added and grown at 20 °C for 20 h. Cells were pelleted by centrifugation and the spent medium was transferred to fresh tubes. A sum of 5 mL hexane was added to the spent medium, vortexed briefly, and centrifuged to separate the phases. The hexane layers were concentrated to 50 µL, and 5 µL was used for gas chromatography-mass spectrometry (GC-MS) analysis. For in planta assay, overnight *A. tumefaciens* cultures were pelleted and resuspended in MMA solution (10 mM MES, 10 mM MgCl_2_, 100 µM acetosyringone) to OD_600_ = 1. Five-weeks-old tobacco leaves were co-infiltrated with the bacterial suspensions harbouring plasmids expressing *35S_pro_:ObEGS* and silencing suppressor *35S_pro_:p19*, together with or without *35S_pro_:ObCAAT2*. The infiltrated plants were incubated in growth chamber with a 16 h photoperiod at 25 °C for 3 d before subjected to volatile collection. Four intact plants per construct were enclosed in a glass cylinder with incoming purified air at 1 L/min, and the volatiles were collected through a cartridge packed with 200 mg HayeSep Q polymer (Hayes Separations Inc., Bandera, TX, USA) at 0.8 L/min air flow as described by [54]. Collections were done in growth chamber with conditions as above for 20 h. The trapped volatiles were eluted with 200 mL hexane containing 10 mg/mL camphor as an internal standard and analysed using GC-MS. Compound identification was done by comparison of the mass spectra and retention times with those from NIST mass spectral library and authentic standards. P19 and ObCAAT2 infiltrated plants were used as negative controls. For additional substrate studies, following 2 days post infiltration with *35S_pro_:ObEGS* and/or *35S_pro_:ObCAAT2*, the leaves were re-infiltrated with *p*-coumaryl alcohol (2 mg/mL) or coniferyl alcohol (1 mg/mL) dissolved in 0.2% EtOH. After drying for 1 h, the plants were immediately set up for headspace sampling.

### 4.9. Elicitor Treatment

Wild type sweet basil seeds were sterilized and allowed to germinate on MS media plates. 45 d after germination the plants were moved to fresh MS plates containing elicitors. Methyl jasmonate (150 µM), gibberellic acid (30 µM) and salicylic acid (200 µM) (Sigma, Singapore) were dissolved in ethanol. Ethylene (50 µM) (Sigma, Singapore) was dissolved in 20% ethanol. Pectinase (720 units/500 mL) (Sigma, Singapore) was dissolved in water. All the solutions were sterilized by passing through a filter (0.22 µm) before adding to the MS media. From the third day onwards, the roots of the plants were harvested each day and subjected to GC. This was repeated till the day eugenol was detected. For bacterial treatment, five isolates of *Bacillus*, *Bacillus cereus*, *Bacillus megaterium*, *Bacillus thuringiensis*, *Bacillus toyonensis* and *Bacillus aryabhattai* were cultured separately. Cells were pelleted and resuspended in water. All five solutions were mixed together. The plant roots were dipped in this solution for 10 s with agitation and placed in fresh MS plates. Seven days post infection (dpi) the roots were subjected to GC-MS. For fungal treatment *Trichoderma viridae* was grown and the spores were scraped off and dissolved in water. The plant roots were dipped in the mixed solution for 10 s with agitation and placed in fresh MS plates. After three dpi the roots were subjected to GC.

### 4.10. GC-MS Analysis

For sweet basil, leaves of 3–4 cm at the fourth node were used and 45 d old soil-grown roots and aseptically-grown roots were used. For *N. benthamiana*, the infiltrated leaves were collected and used for GC-MS analysis. Diethyl sebacate and camphor were added as internal standards in sweet basil, and tobacco samples, respectively. Homogenised samples in 500 µL ethyl acetate were incubated for 10 min at room temperature with vigorous shaking followed by a centrifugation for 10 min at 13,000 revolutions per minute (rpm). The top layer was transferred to new tube. Anhydrous Na_2_SO_4_ was used to dehydrate the collected organic layer. The samples were analysed using GC-MS (7890A with 5975C inert MSD with triple axis detector, Agilent Technologies, Santa Clara, CA, USA). 2-5 µL of samples were injected and separation was achieved with a temperature program of 50 °C for 1 min and increased at a rate of 8 °C/min to 300 °C and held for 5 min, on a 30 m HP-5 MS column (Agilent Technologies, Santa Clara, CA, USA).

Glycosylated eugenol was extracted as described previously [37] with minor modifications. 300 mg of tissue were ground in liquid nitrogen and homogenized with 1.2 mL of 80% methanol. The homogenate was then sonicated for 20 min and later centrifuged at 16,000 g for 10 min. The supernatant was vacuum dried and resuspended in 0.9 mL of 0.15 M citrate-phosphate buffer, pH 5.4. To the buffer, 150 µL of Viscozyme and 140 units of β-glucosidases were added and incubated at 37 °C for overnight. On the next day, 800 µL of hexane was added to the samples and incubated at room temperature on a shaking platform at 150 rpm. The samples were then centrifuged at 10,000 g for 10 min and the supernatant was concentrated to 50 µL. The aglycones were then analysed by GC-MS as described above.

### 4.11. HPLC Analysis

For detecting Rosmarinic acid, Shidmadzu Nexera X2 UHPLC system with a photodiode array detector (SPD-M30A with high sensitivity cell) and XDB C-18 column was used. Roots were freeze dried in VirTis vacuum dryer (SP scientific, Ipswich, UK) for 48 h following which 200 mg of tissue was homogenized in 10 mL of 60% ethanol. The tubes were then placed in a water bath and sonicated for 15 min at 25 °C and later centrifuged at 5000 rpm for 15 min. The supernatant was filtered and 5 µL was injected for analysis. The parameters for HPLC analysis was followed as described previously [55]. RA peak in the samples was identified by comparing the retention time with that of a commercial RA standard (Sigma). Integrated peak area was compared with RA standard calibration curve for RA quantification in the samples. Data are indicated as “mean ± SE” of three biological replicates each performed in triplicates.

### 4.12. Total Protein Isolation

The total protein was isolated from 45 d old roots of plants grown in soil/aseptic conditions. A sum of 2 g of roots were grinded to powder using liquid nitrogen and homogenized in 4 mL of buffer (50 mM Tris [pH 7.8], 10 mM β-mercaptoethanol, 1 mM phenylmethylsulfonyl fluoride, protease inhibitor [10 µL/mL] and phosphatase inhibitor [only for samples used for phosphoprotein enrichment]). The homogenate was kept is ice for 30 min followed by centrifugation at 15,000 rpm for 10 min at 4 °C. The supernatant was then re-centrifuged using centrifuge filters at 15,000 rpm for 2 min and flow through was stored for further analysis. The total amount of protein in the flow through was estimated using Bradford reagent and equal amounts were then used for western analysis, phosphoprotein enrichment and ubiquitin enrichment.

### 4.13. Phosphoprotein Enrichment and Ubiquitin Enrichment

Phosphoprotein and ubiquitin were enriched using the Pierce™ Phosphoprotein Enrichment kit and the Pierce™ Ubiquitin Enrichment kit (Thermo Fisher Scientific, Singapore) according to the manufacture’s protocol. The enriched proteins were then used for western analysis.

### 4.14. Western Blot Analysis

Western blot analysis was pursued as described previously [56] with minor modifications. The total protein, phosphoprotein, ubiquitin enriched protein or pellet of 1ml of IPTG induced recombinant ObEGSs culture were mixed with Laemmli sample buffer and incubated at 100 °C for 5 min. The samples were then cooled and loaded into gel. Mini-PROTEAN Precast Gels from Bio-Rad were used for gel run at 200 V for 35 min. The gel was later transferred to polyvinylidene difluoride membrane for overnight at 4 °C. On the next day, blocking, the primary antibody (ObEGS4) and secondary antibody (anti-mouse with HRP conjugate) incubation was pursued as described previously [56]. Primary antibody (Genscript, Singapore) was used at 1:500 dilution and secondary antibody (GE Healthcare, Singapore) at 1:2000 dilution. The blot was developed using Clarity Western ECL (Bio-Rad, Singapore) substrates and ChemiDoc™ Touch Imaging System from Bio-Rad (Singapore).

### 4.15. Phylogenetic Analysis

Phylogenetic tree was constructed using MEGA7 software by Neighbour-joining method with bootstrap values of 1000 replicates. Sequences used for generating the tree were obtained from NCBI database, accession numbers of which are listed in Supplementary Table S3.

### 4.16. Statistical Analysis

Data are indicated as “mean ± SE” of three biological replicates each performed in triplicates.

## Data Availability

The data presented in this study are openly available in GenBank (https://www.ncbi.nlm.nih.gov/genbank/). Sequence data of *ObEGS2*, *ObEGS3*, *ObEGS4*, *ObEGS5*, *ObEGS6*, *ObEGS7* and *ObEGS8* have been deposited in GenBank under the accession numbers MN686216, MN686217, MN686218, MT762127, MT762128, MT762129 and MT762130. Sequence data of *ObCAAT2* has been deposited in GenBank previously under the accession number MN031889. RNA sequencing data of PGT, leaf-PGT, leaf and root tissues of sweet basil can be accessed from NCBI SRA under the Bioproject number PRJNA54736.

## Supplementary Materials

The following are available online at https://www.mdpi.com/1422-0067/22/2/681/s1, Figure S1. Schematic diagram showing phenylpropanoid pathway, Figure S2. NetPhos 3.1 result, Figure S3. Expression levels of *ObCAAT2* and *ObEGSs*, Table S1. List of primers used in this study, Table S2. Identity percentage of protein sequences of ObEGSs, Table S3. NCBI accession numbers of sequences used in phylogenetic tree construction in Figure 3, Table S4. List of elements present in *ObEGS1* promoter sequence, Table S5. List of elements present in *ObEGS4* promoter sequence.
