# Supplementary material for: Sweet Basil Has Distinct Synthases for Eugenol Biosynthesis in Glandular Trichomes and Roots with Different Regulatory Mechanisms
Source: Int J Mol Sci. 2021 Jan 12;22(2):681. doi: 10.3390/ijms22020681 (PMC7826958; doi:10.3390/ijms22020681)
Supplement: Supplementary file 1 [file ijms-22-00681-s001.pdf]

Title: Sweet basil has distinct synthases for eugenol biosynthesis in glandular trichomes and roots with different regulatory mechanisms

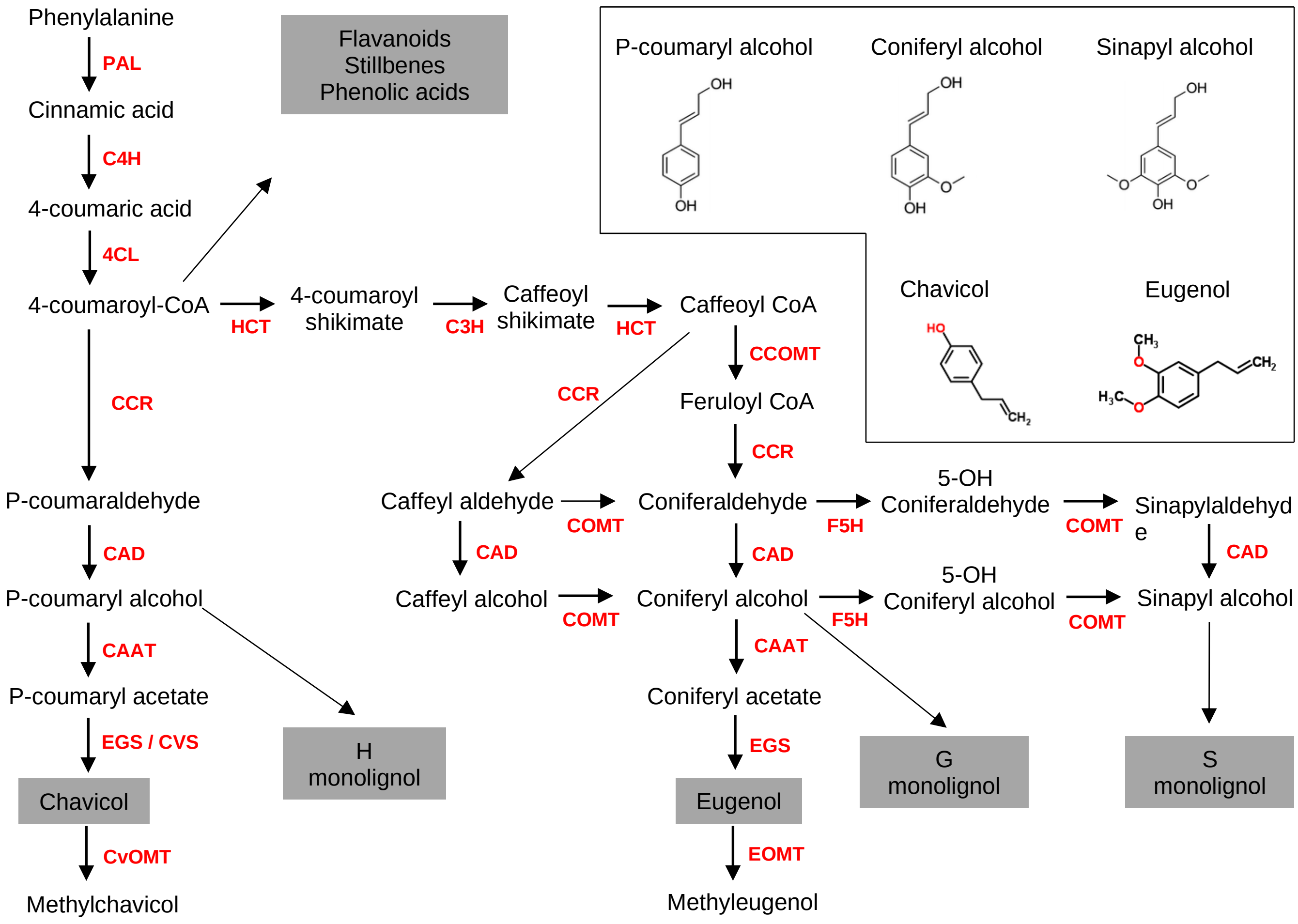

Supplementary Figure S1. Schematic diagram showing phenylpropanoid pathway. Products of the phenylpropanoid pathway are highlighted with grey boxes. PAL, phenylalanine ammonia lyase; C4H, cinnamate 4-hydroxylase; 4CL, 4-coumaroyl CoAligase; CCR, cinnamoyl-CoA reductase; CAD, cinnamyl alcohol dehydrogenase; CAAT, coumaryl alcohol acetyl transferase; CVS, chavicol synthase; CvOMT, chavicol O-methyltransferase; EGS, eugenol synthase; EOMT, eugenol O-methyltransferase; HCT, hydroxycinnamoyl-CoA shikimate/quinate hydroxycinnamoyl transferase; C3H, p-coumaroyl shikimate 3'-hydroxylase; CCOMT, caffeoyl-CoA O-methyltransferase; COMT, caffeic acid O-methyltransferase; F5H, ferulate-5-hydroxylase.

A

```
>seq.1  310 amino acids
#
# netphos-3.1b prediction results
#
# Sequence                   # x   Context     Score   Kinase    Answer
# -------------------------------------------------------------------
# seq.1                     24 S   IVEASAKSG   0.914   unsp       YES
#
# seq.1                     27 S   ASAKSGHPT   0.978   unsp       YES
#
# seq.1                     38 S   LLRESAVSD   0.938   unsp       YES
#
# seq.1                    185 T   YGDGTAKAV   0.903   unsp       YES
#
# seq.1                    203 S   YTIKSVDDP   0.997   unsp       YES
#
# seq.1                    294 Y   ASELYPDVK   0.950   unsp       YES
#
# seq.1                    299 Y   PDVKYTTVE   0.909   unsp       YES
#
    MAEKSKILIIGGTGYIGKFIVEASAKSGHPTFALLRESAVSDPAKSQLIE   #    50
    GFKSSGVTILIGDLNDYESLVKAVKQVDVVISTVGALQLGDQKNIIAAIK   #   100
    EAGNIKRFYPSEFGNDVDRTRAVEPAKSAFAEKAKIRRAIEASGIPYTFV   #   150
    SSNYFAGYSLPTLSQPGLFAPPPPKDKIFIYGDGTAKAVFNDEHDIGTYT   #   200
    IKSVDDPRTVNKVMYIKPPKNIYSFNELVALWEKKIGKTVEKEYLPEDKL   #   250
    LKMIEESPIPVNVILAINHSTYVKGDQTYFEIDPSVAVEASELYPDVKYT   #   300
    TVEEYLSNFV                                           #   350
%1  .......................S..S..........S............   #    50
%1  ..................................................   #   100
%1  ..................................................   #   150
%1  ..................................T...............   #   200
%1  ..S...............................................   #   250
%1  ...........................................Y....Y.   #   300
%1  ..........
```

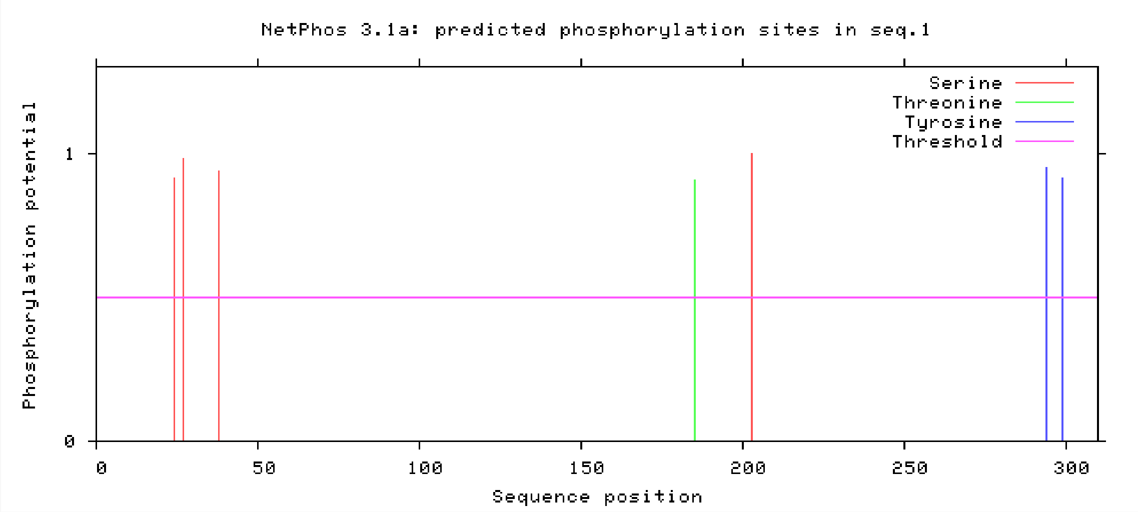

B

```
>Sequence       314 amino acids
#
# netphos-3.1b prediction results
#
# Sequence                  # x   Context     Score   Kinase    Answer
# -------------------------------------------------------------------
# Sequence                 42 S   TRPNSSKTT   0.996   unsp        YES
#
    MEENGMKSKILIFGGTGYIGNHMVKGSLKLGHPTYVFTRPNSSKTTLLDE   #     50
    FQSLGAIIVKGELDEHEKLVELMKKVDVVISALAFPQILDQFKILEAIKV   #    100
    AGNIKRFLPSDFGVEEDRINALPPFEALIERKRMIRRAIEEANIPYTYVS   #    150
    ANCFASYFINYLLRPYDPKDEITVYGTGEAKFAMNYERDIGLYTIKVATD   #    200
    PRALNRVVIYRPSTNIITQLELISRWEKKIGKKFKKIHVPEEEIVALTKE   #    250
    LPEPENIPIAILHCLFIDGATMSYDFKENDVEASTLYPELKFTTIDELLD   #    300
    IFVHDPPPPASAAF                                       #    350
%1  .........................................S........   #     50
%1  ..................................................   #    100
%1  ..................................................   #    150
%1  ..................................................   #    200
%1  ..................................................   #    250
%1  ..................................................   #    300
%1  ..............
```

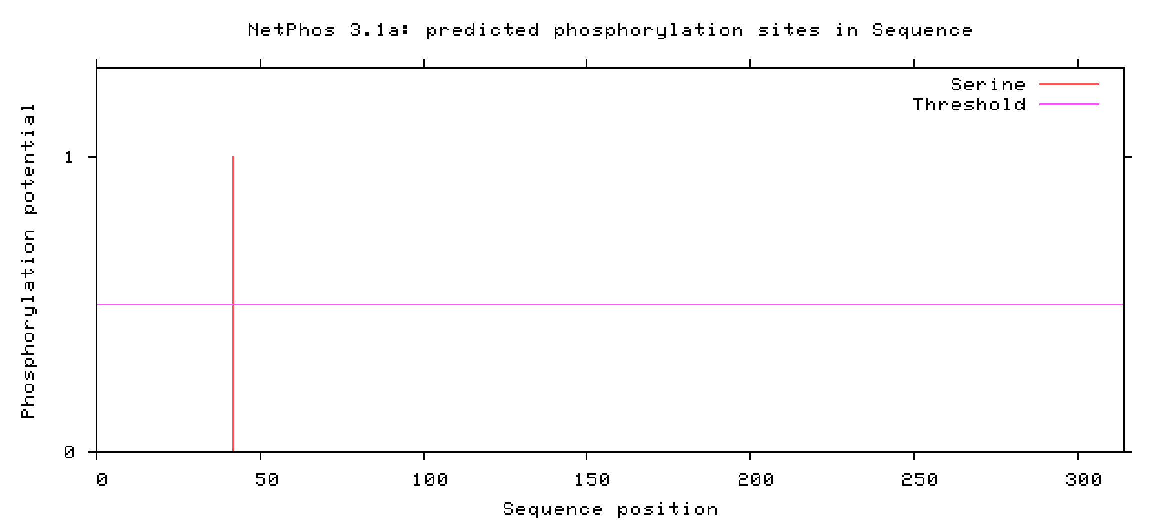

Supplementary Figure S2. NetPhos 3.1 result. Predicted potential sites for phosphorylation in (A) ObEGS4 protein and (B) ObEGS1 protein

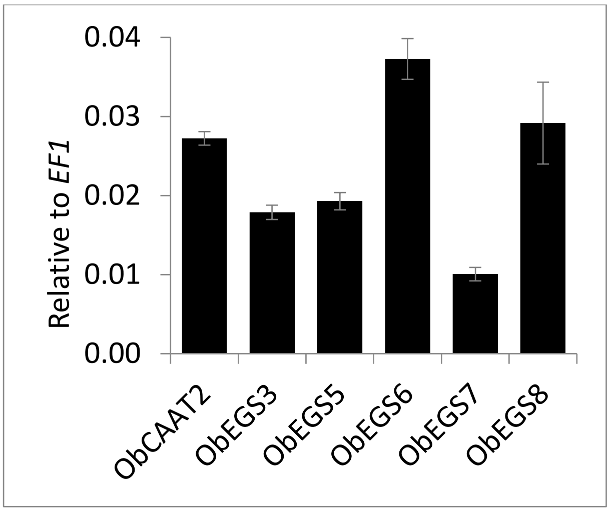

Supplementary Figure S3. Expression levels of *ObCAAT2* and *ObEGSs*. Expression levels of *ObCAAT2*, *ObEGS*3, *ObEGS*5, *ObEGS*6, *ObEGS*7 and *ObEGS*8 in 45 d old aseptically-grown roots.

Supplementary Table S1. List of primers used in this study

**Name**	**Sequence (5' to 3')**	**Purpose**
B_EGS1_NL_F	CACCATGGAGGAAAATGGGATGAAAAGC	Subcellular localization
B_EGS1_NL_R	AAATGCTGCTGAAGCCGGCG	Subcellular localization
B_EGS2_NL_F	CACCATGGGTGAGAGAAGCAAAATATT	Subcellular localization
B_EGS2_NL_R	TGCGAAAGTTGCCAAAGTTGG	Subcellular localization
B_EGS3_NL_F	CACCATGGCAGAAAAGAAGAGCAAG	Subcellular localization
B_EGS3_NL_R	GATGAACTGGTTGAGGTAGTCATCAACAG	Subcellular localization
B_EGS4_NL_F	CACCATGGCGGAGAAGAGCAAGATT	Subcellular localization
B_EGS4_NL_R	GACAAAGTTGCTGAGGTATTCCTCCAC	Subcellular localization
B_EGS5_NL_F	CACCATGGTAGTAGATAATAAGATTAG	Subcellular localization
B_EGS5_NL_R	CTCAAAAGCTGCCGCCGCGGGCTC	Subcellular localization
B_EGS6_NL_F	CACCATGGCAGAGAAGATTTTGATCATC	Subcellular localization
B_EGS6_NL_R	CACAAAGTTATTGAGGTACT	Subcellular localization
B_EGS7_NL_F	CACCATGGCCGGAAAGAGTAAGATTT	Subcellular localization
B_EGS7_NL_R	AACATACTGATCAAGCATCTC	Subcellular localization
B_EGS8_NL_F	CACCATGGCAAAAAGCAAAGTTCTAA	Subcellular localization
B_EGS8_NL_R	CACATATCGTTTAAGGTATTC	Subcellular localization
B_EGS1_F	CACCATGGAGGAAAATGGGATGAAA	expression
B_EGS1_R	TTAAAATGCTGCTGAAGC	expression
B_EGS2_F	CACCATGGGTGAGAGAAGCAAAATATT	expression
B_EGS2_R	TCATGCGAAAGTTGCCAAAGTTG	expression
B_EGS3_F	CACCATGGCAGAAAAGAAGAGCAAG	expression

B_EGS3_R	TTAGATGAACTGGTTGAGGTAGTCATCAAC	expression
B_EGS4_F	CACCATGGCGGAGAAGAGCAAGATT	expression
B_EGS4_R	TCAGACAAAGTTGCTGAGGTATTCCTCCAC	expression
B_EGS5_F	CACCATGGTAGTAGATAATAAGATTAG	expression
B_EGS5_R	TCACTCAAAAGCTGCCGCCGCGGGCTC	expression
B_EGS6_F	CACCATGGCAGAGAAGATTTTGATCATC	expression
B_EGS6_R	CTACACAAAGTTATTGAGGTACT	expression
B_EGS7_F	CACCATGGCCGGAAAGAGTAAGATTT	expression
B_EGS7_R	TCAAACATACTGATCAAGCATCTC	expression
B_EGS8_F	CACCATGGCAAAAAGCAAAGTTCTAA	expression
B_EGS8_R	CTACACATATCGTTTAAGGTATTC	expression
B_CAAT_F	CACCATGTATATTTTCCCACCTCCA	expression
B_CAAT_R	CTAAGGTAGGTTGAAAGAGTGAAATGAGA	expression
EGS5-R-race1	ACAGTGATCTCATCTTTTTCCTCAGAAG	RACE
EGS5-R-nst1	TGAAATATGCAGCGTAGCAATTTGCAG	RACE
EGS6-R-race1	TTGAACACAGCTTTGACATTGCCGTCG	RACE
EGS6-R-nest1	GTGGCGCCCATCTGCAGCATGTTGGAG	RACE
EGS8-R-race1	ATAGCCGGCGACACAGTTTGCAGAGAC	RACE
EGS8-R-nest1	AGCTTCTTCAATGGCTTTCCTCACCAC	RACE
EGS8-F-race1	TGTCGTCATACATGGAGACGGCAATAC	RACE
EGS8-F-nest1	ATGACCTAGCCACTTATACCATAAAGAC	RACE
GW_EGS1_GSP1	CCCAAGGATTGGAACTCATCAAGAAGGGTTGTC	Genome walking

GW_EGS1_GSP2	CACCATGTGATTTCCAATGTAACCTGTCCCTCC	Genome walking
GW_EGS4_GSP1	GTGGGGTGGCCGGATTTAGCACTGGCTTCC	Genome walking
GW_EGS4_GSP2	CCACCAATGATGAGAATCTTGCTCTTCTCCGC	Genome walking
ObEF- F	AATGGCAAAAAGCTCGAAGA	qPCR
ObEF- R	TCGCAGACATGACAGACACA	qPCR
q_EGS1-F	GGTGCCATCATAGTCAAGGG	qPCR
q_EGS1-R	GCAATGCGTTTATTCTGTCCT	qPCR
q_EGS2-F	CAATCACCTCACCCTCACCG	qPCR
q_EGS2-R	TTCTCCCAGGAAGCAACCAA	qPCR
q_EGS3-F	CGTCGGAGTTTGGTTGTGAT	qPCR
q_EGS3-R	GAGGCTCAGTTCTATTAGGTTGC	qPCR
q_EGS4-F	CGCGCTGCAGTTGGGAGATC	qPCR
q_EGS4-R	AGAGGCCAGGCTGGGACAG	qPCR
q_EGS5-F	CACCGATCCGAGAACCTGCAACCGG	qPCR
q_EGS5-R	CGAAGCCTCGACGTCATCGCCCTCG	qPCR
q_EGS6-F	CAACGACGAACGCGACATTGGGGCATAC	qPCR
q_EGS6-R	CACCGGAATCGGCGACTCTTGG	qPCR
q_EGS7-F	GACCGTGTCCATGCTGTTGAGCCG	qPCR
q_EGS7-R	TGGGGTAACTGTGGCGTCTAACTGA	qPCR
q_EGS8-F	AGAGCCTCGTGGACGCCGT	qPCR
q_EGS8-R	CGCCGTCGCCAATGCCTTC	qPCR

P_EGS1_F	CACCACAGGCTATAGTAGCAACAAATTGG	Promoter expression
P_EGS1_R	CTTGCTTTTCTACACAACAACTCTTAAAGG	Promoter expression
P_EGS4_F	CACCTGCTCATCACACCTCACTCACAAGTA	Promoter expression
P_EGS4_R	CCTTCCTTTGTTTTGCTTCCTGCGA	Promoter expression

Supplementary Table S2. Identity percentage of protein sequences of ObEGSs.

	**ObEGS2**	**ObEGS3**	**ObEGS4**	**ObEGS5**	**ObEGS6**	**ObEGS7**	**ObEGS8**
**ObEGS1**	52.4	42.8	46.0	66.8	46.3	41.7	41.8
**ObEGS2**	-	38.0	42.3	46.5	39.7	37.1	37.3
**ObEGS3**	-	-	58.2	43.2	58.9	59.5	43.8
**ObEGS4**	-	-	-	47.3	72.2	63.0	46.5
**ObEGS5**	-	-	-	-	48.7	46.0	42.8
**ObEGS6**	-	-	-	-	-	62.4	48.0
**ObEGS7**	-	-	-	-	-	-	46.2

Supplementary Table S3. NCBI accession numbers of sequences used in phylogenetic tree construction in Figure 3

GENE NAME	NCBI ACCESSION NUMBER
ObEGS1 - Ocimum basilicum eugenol synthase1	ABD17321.1
ObEGS2 - Ocimum basilicum eugenol synthase2	MN686216
ObEGS3 - Ocimum basilicum eugenol synthase3	MN686217
ObEGS4 - Ocimum basilicum eugenol synthase4	MN686218
ObEGS5 - Ocimum basilicum eugenol synthase5	MT762127
ObEGS6 - Ocimum basilicum eugenol synthase6	MT762128
ObEGS7 - Ocimum basilicum eugenol synthase7	MT762129
ObEGS8 - Ocimum basilicum eugenol synthase8	MT762130
Clarkia breweri isoeugenol synthase1	ABR24112.1
Clarkia breweri eugenol synthase1	ABR24113.1
Clarkia breweri eugenol synthase2	ABR24114.1
Pimpinella anisum t anol/ isoeugenol synthase	ACL13526.1
Petunia hybrida isoeugenol synthase1	ABD17322.1
Petunia hybrida eugenol synthase1	ABR24115.1
Ocimum gratissimum isolate I eugenol synthase	AOC97441.1
Ocimum kilimandscharicum eugenol synthase	AOC97443.1
Ocimum tenuiflorum eugenol synthase	AOC97445.1
Ocimum americanum eugenol synthase	AOC97450.1
Rosa chinensis eugenol synthase	AFQ98278.1
Gymnadenia odoratissima eugenol synthase 1	AKB11751.1
Gymnadenia odoratissima eugenol synthase 2	AKB11752.1

Gymnadenia conopsea eugenol synthase 1	AKB11747.1
Gymnadenia conopsea eugenol synthase 2	AKB11748.1
Gymnadenia densiflora (iso)eugenol synthase 1	AKB11749.1
Gymnadenia densiflora (iso)eugenol synthase 2	AKB11750.1
Glycine soja eugenol synthase 1	KHN37444.1
Glycine soja isoeugenol synthase 1	KHN26455.1
Cajanus cajan Eugenol synthase 1	KYP30999.1
Theobroma cacao isoeugenol synthase 1	EOY25510.1
Larrea tridentate allylphenol synthase 1	AHA90804.1
Larrea tridentate allylphenol synthase 2	AHA90805.1
Larrea tridentate propenylphenol synthase 1	AHA90806.1
Piper regnellii allylphenol synthase 1	AHA90807.1
Piper regnellii allylphenol synthase 2	AHA90808.1
Piper regnellii propenylphenol synthase 1	AHA90809.1
Daucus carota eugenol/isoeugenol synthase 1	XP_017241251.1
Gymnadenia densiflora eugenol/isoeugenol synthase 1	AKB11749.1
Fragaria ananassa eugenol synathse 2	AGV02008.1

Supplementary Table S4. List of elements present in *ObEGS1* promoter sequence

**Element**	**Function**
AC-II	Known to be bound by R2R3-MYBs
Box 4	part of a conserved DNA module involved in light responsiveness
Box 1, Sp1	light responsive element
LAMP-element	part of a light responsive element
circadian	*cis*-acting regulatory element involved in circadian control
TC-rich repeats	*cis*-acting element involved in defence and stress responsiveness
Skn-1_motif	*cis*-acting regulatory element required for endosperm expression
Box II -like sequence	*cis*-acting regulatory element
HSE	*cis*-acting element involved in heat stress responsiveness
TCA-element	*cis*-acting element involved in salicylic acid responsiveness

Supplementary Table S5. List of elements present in *ObEGS4* promoter sequence

**Element**	**Function**
MBS	MYB binding site involved in drought-inducibility
Box 4	part of a conserved DNA module involved in light responsiveness
Box 1, GT1-motif	light responsive element
ACE	*cis*-acting element involved in light responsiveness
AE-box	part of a module for light response
G-box	*cis*-acting regulatory element involved in light responsiveness
GAG-motif, TCT-motif, CATT-motif, LAMP-element	part of a light responsive element
OBP-1 site	*cis*-acting regulatory element
TC-rich repeats	*cis*-acting element involved in defence and stress responsiveness
Skn-1_motif	*cis*-acting regulatory element required for endosperm expression
ARE	*cis*-acting regulatory element essential for the anaerobic induction
HSE	*cis*-acting element involved in heat stress responsiveness
CGTCA-motif, TGACG-motif	*cis*-acting regulatory element involved in the MeJA-responsiveness

GARE-motif	gibberellin-responsive element
ERE	ethylene-responsive element
O2-site	*cis*-acting regulatory element involved in zein metabolism regulation
LTR	*cis*-acting element involved in low-temperature responsiveness
WUN-motif	wound-responsive element
